# Biochar particle size, shape, and porosity act together to influence soil water properties

**DOI:** 10.1371/journal.pone.0179079

**Published:** 2017-06-09

**Authors:** Zuolin Liu, Brandon Dugan, Caroline A. Masiello, Helge M. Gonnermann

**Affiliations:** 1 Department of Earth Science, Rice University, MS, Houston, Texas, United States of America; 2 Departments of Chemistry and BioSciences, Rice University, MS, Houston, Texas, United States of America; RMIT University, AUSTRALIA

## Abstract

Many studies report that, under some circumstances, amending soil with biochar can improve field capacity and plant-available water. However, little is known about the mechanisms that control these improvements, making it challenging to predict when biochar will improve soil water properties. To develop a conceptual model explaining biochar’s effects on soil hydrologic processes, we conducted a series of well constrained laboratory experiments using a sand matrix to test the effects of biochar particle size and porosity on soil water retention curves. We showed that biochar particle size affects soil water storage through changing pore space between particles (interpores) and by adding pores that are part of the biochar (intrapores). We used these experimental results to better understand how biochar intrapores and biochar particle shape control the observed changes in water retention when capillary pressure is the main component of soil water potential. We propose that biochar’s intrapores increase water content of biochar-sand mixtures when soils are drier. When biochar-sand mixtures are wetter, biochar particles’ elongated shape disrupts the packing of grains in the sandy matrix, increasing the volume between grains (interpores) available for water storage. These results imply that biochars with a high intraporosity and irregular shapes will most effectively increase water storage in coarse soils.

## Introduction

Biochar is charcoal made for the purpose of soil amendment [1]. Amending soil with biochar is an approach to mitigate climate change [2] and to improve crop productivity [1, 3]. Once mixed with soil, biochar can affect plant growth by altering soil hydrologic properties [4–7] and nutrient availability [8].

Water movement and storage in soils are crucial for nutrient delivery and plant productivity. Biochar has the potential to alter soil hydrology and to drive shifts in the amount of water stored in soils. To understand how biochar amendment may influence water delivery to plants, we must understand how biochar affects soil hydrologic processes. However, while many studies report effects of specific biochars on specific soil water properties [9, 10], there is a dearth of mechanistic information available, and mechanisms are needed to predict under what circumstances biochar will have beneficial effects on soils.

Understanding how the amount of water held at field capacity (*θ*_*fc*_) and at permanent wilting point (*θ*_*pwp*_), and the amount of plant available water (*θ*_*paw*_) of soil change with biochar amendment is an efficient way to quantify how biochar affects soil water conditions and plant growth. We use the water retention curve, which defines equilibrium water content (*θ*) at a given soil water potential (*ψ*), to extract the key parameters of water content at saturation (*θ*_*s*_), *θ*_*fc*_ (water content at *ψ* = -33kPa), *θ*_*pwp*_ (water content at *ψ* = -1500kPa), and *θ*_*paw*_ (= *θ*_*fc*_ - *θ*_*pwp*_) [11, 12]. Water held at field capacity is also defined as the water present in the soil after gravity-driven drainage. Water held at and beyond permanent wilting point is assumed to be held at a pressure too high for plants to extract from soil [13].

Previous studies have shown that biochar increased water retention of soil [14]; however, the mechanisms controlling these observations remain elusive. Sandy soils are a particularly appealing target for biochar amendment because studies on sand and sandy loam often show an increase in *θ*_*paw*_ after biochar amendment [10, 15–18]. However, few studies focused on the mechanism of how biochar increase *θ*_*paw*_. Without understanding the mechanisms that control biochar-driven changes of water retention of soil, it is difficult to predict when and by how much biochar will improve soil water retention.

Biochar’s particle size, shape, and internal structure likely play important roles in controlling soil water storage because they alter pore characteristics. For instance, biochar has pores inside of particles (intrapores), which may provide additional space for water storage beyond the pore space between particles (interpores) [19]. Particle size may influence both intrapores and interpores through different processes because the size and connectivity of these particles likely differ. In addition, when applied in the field, biochar particles may have different sizes and shapes compared to soil particles. This addition of biochar grains with different shapes and sizes will change interpore characteristics (size, shape, connectivity, and volume) of soil and thus will affect water storage and mobility. For instance, fine biochar particles can fill pores between coarse soil particles, decreasing pore size and changing interpore shape. Conversely, high aspect ratio biochar particles may interfere with packing of low aspect ratio soil grains, leading to increased interpore sizes. Both of these cases can be expected to change soil water retention.

Here we develop a mechanistic understanding of how and when biochar application affects water retention in a sandy matrix. Sandy matrices are a particularly important system to constrain because biochar application has the potential to increase the resilience of agriculture in sandy systems. We determined *θ*_*s*_, *θ*_*fc*_, *θ*_*pwp*_, and *θ*_*paw*_ by measuring water retention curves of sand mixed with three particle-size ranges of biochar at 2 wt% (kg biochar/kg total dry mixture x100%). In addition, we conducted control experiments measuring water retention curves of sand plus fine sand (<0.251 mm, volume of fine sand was equal to volume of fine biochar at 2 wt% biochar rate) and sand plus coarse sand (0.853–2.00 mm, volume of coarse sand was equal to volume of coarse biochar at 2 wt% biochar rate). We constrained a suite of physical properties (skeletal density, envelope density, and biochar intraporosity) that influence water retention. Last, we qualitatively and quantitatively characterized the size and shape of biochar particles. Using these constraints, we develop a conceptual model of how these physical properties drive changes in water retention of biochar-sand mixtures.

## Materials and methods

### Sample preparation

We pyrolyzed mesquite feedstock (2.00–2.30 mm) at 400°C for 4 hours to form biochar (Table 1) as described in Kinney et al. [20] and Liu et al. [21]. Ash content, pH, electrical conductivity, and carbon, nitrogen, and hydrogen content of biochar were reported in Liu et al. [21]. We ground and sieved biochar into three sizes: fine (<0.251 mm, NO. 60 U.S. Std. mesh), medium (0.251–0.853 mm) and coarse (0.853–2.00 mm, NO. 20-NO. 10 U.S. Std. mesh). To obtain accurate mass fractions, all sand and biochar were oven dried at 60°C for 72 hours to remove any water absorbed during storage. We then mixed 2 wt% biochar into the sand (silica sand [Pavestone, US] sieved into 0.251–0.853 mm, NO. 60-NO. 20 U.S. Std. mesh) (Table 1). We created controls by mixing medium sand with fine sand (<0.251 mm, volume of fine sand was equal to volume of fine biochar at 2 wt% biochar rate) and coarse sand (0.853–2.00 mm, volume of coarse sand was equal to volume of coarse biochar at 2 wt% biochar rate).

**Table 1:** Descriptions of silica sand and biochar used in this study.

Properties	Data
*Silica sand*	
Sieved particle size (mm)	<0.251, 0.251–0.853, 0.853–2.00
*Biochar*	
Feedstock	Mesquite
Heating rate (˚C/min)	5
Heating duration (hours)	4
Pyrolysis temperature (°C)	400
Feedstock particle size before pyrolysis (mm)	2.00–2.30
Biochar sieved particle size used in experiment (mm)	<0.251, 0.251–0.853, 0.853–2.00
Biochar rate (wt%)	2
Ash Content (wt%)	4.26
pH	7.41
Electric Conductivity (μS/cm)	110
C %	73.0 ± 0.4
H %	3.2 ± 0.1
N %	0.74 ± 0.01

To understand pore systems and water storage, we measured the skeletal density (density of the solids without intrapores, *ρ*_*s*_) of biochar and sand by helium pycnometry in a 1cm^3^ sample chamber (AccuPyc II 1340, Micromeritics, Norcross, GA) and the envelope density of biochar (density including intrapores, *ρ*_*e*_) (Geopyc 1360, Micromeritics, Norcross, GA) (Table 2). AccuPyc measures biochar’s skeletal volume by detecting the pressure change due to the change of helium volume that is displaced by biochar’s skeleton. It is assumed that helium molecules penetrate all biochar intrapores. Skeletal density was then obtained using biochar mass divided by its skeletal volume. Geopyc measures biochar’s envelope volume by subtracting volume of a consolidated quasi-fluid composed of small, rigid spheres (DryFlo) from the volume of the same consolidated DryFlo after biochar has been added. Enveloped density is the result of biochar mass divided by the envelope volume. We only measured *ρ*_*e*_ of biochar before grinding due to instrumental limitation on the minimum particle size measurable. However, grinding biochar into smaller size may result in reduction of intraporosity and thus may affect water storage. For details on measurement of *ρ*_*s*_ and *ρ*_*e*_, see Brewer et al. [22]. We then used these two measurements to calculate porosity of biochars (*ϕ*_*b*_ = 1 - *ρ*_*e*_/*ρ*_*s*_). The use of *ρ*_*s*_ and *ρ*_*e*_ measurements to calculate porosity compares favorably with porosity determined by mercury (Hg) porosimetry and with N_2_-sorption based techniques (e.g. BET). Benefits to density-based porosity measurements include ease, speed, low cost, and no involvement of toxic materials. Like Hg porosimetry, density-based measurements detect the entire range of pore sizes in a sample, compared to N_2_-sorption based techniques, which can only detect very small pores and can miss >90% of the pore volume of biochars [22]. In addition, while Hg porosimetry measures total porosity (pores inside plus pores between particles), the combined total porosity measured through density analysis measures only the porosity inside of particles (intrapores) and does not detect the porosity between particles (interpores). Two disadvantages of density-based techniques include: (a) the inability to make measurements on particles smaller than 2 mm, requiring us to assume that the porosity of small biochar was approximately equal to that of the large biochar; (b) density-based porosity measurements provide only total porosity, unlike Hg porosimetry, which can provide information on the entire spectrum of pore characteristics.

**Table 2:** Particle size, skeletal density (*ρ*_*s*_), and envelope density (*ρ*_*e*_) of sand and biochar used in this study. We report average and standard deviation of at least three measurements.

Materials	Sieved particle size (mm)	*ρ*_*s*_ (kg/m^3^)	*ρ*_*e*_ (kg/m^3^)
Sand	0.251–0.853	2660 ± 20	
Parent biochar	2.00–2.30	1430 ± 10	570 ± 70
Fine biochar	<0.251	1500 ± 20	
Medium biochar	0.251–0.853	1480 ± 10	
Coarse biochar	0.853–2.00	1450 ± 10	

### Measurement of water retention curves

We measured water retention curves at room temperature (22. 3 ± 0.2°C) using a Hyprop for *ψ* of +2 to -440 kPa and a WP4C device for *ψ* of -100 to -300,000 kPa (both pieces of equipment were made by Decagon Devices Inc., Pullman, WA).

For Hyprop measurements, each sample was poured without intentional compaction into a stainless-steel cylinder (2.5 x 10^−4^ m^3^ volume, diameter 8cm, height 5cm) with a piece of fabric filter and a plastic cap on the bottom. We then put the cylinder with the sample into a beaker with de-aired, purified water (18.2 MΩ-cm, PURELAB® Ultra Laboratory Water Purification Systems, SIMENS, Germany). The use of purified water allowed us to exclude osmotic potential effects from the measurement. In the beaker, purified water rose from the bottom into the sample through a fabric filter and pushed air out of the sample through the top. We considered samples saturated by this technique after at least 24 hours [23]. We installed two tensiometers with heights of 0.5 and 3.5 cm and the cylinder with sample was clamped to the tensiometer assembly. We then removed the fabric filter and the plastic cap to allow water to evaporate from the sample. We monitored *ψ* during evaporation using the Hyprop tensiometers and sample mass by a mass balance (Kern EG 2200, Balingen, Germany) [24]. The water retention curve was defined by the average *ψ* measured by two tensiometers and the water content by volume [*θ = (M*-*M*_*d*_*)V/ρ*_*w*_, m^3^/m^3^] calculated from sample mass with water (*M*, kg), dry sample mass (*M*_*d*_, kg), sample volume (V = 2.5 x 10^−4^ m^3^) and water density (*ρ*_*w*_ = 1000 kg/m^3^).

For biochar-sand mixtures and sand samples, we measured three water retention curves by the Hyprop (S1 Fig). We reported the average and the standard deviation of these replicates. From the Hyprop data, we extracted *θ*_*s*_ and *θ*_*fc*_ and reported the average and standard deviation of these replicated measurements (Table 3). In addition, we measured three replicated water retention curves for fine sand-sand mixtures and coarse sand-sand mixtures using the Hyprop and reported the average and the standard deviation of the replicates.

**Table 3:** Bulk density (*ρ*_*b*_), total porosity (*ϕ*_*T*_), and saturated water content (*θ*_*s*_) of samples for measuring water retention curves by the Hyprop device. We report average and standard deviation of at least three measurements.

Samples	*ρ*_*b*_ (kg/m^3^)	*ϕ*_*T*_ (m^3^/m^3^)	*θ*_*s*_ (m3/m3)
Fine biochar + sand	1500 ± 30	0.47 ± 0.01	0.39 ± 0.03
Medium biochar + sand	1490 ± 20	0.47 ± 0.01	0.41 ± 0.01
Coarse biochar + sand	1480 ± 10	0.47 ± 0.0	0.37 ± 0.04
Sand	1520 ± 20	0.43 ± 0.01	0.34 ± 0.02
Fine sand + sand	1600 ± 00	0.40 ± 0.0	0.36 ± 0.0
Coarse sand + sand	1580 ± 30	0.41 ± 0.01	0.37 ± 0.01

For WP4C measurements we added de-aired, purified water to sand and biochar-sand mixtures to make samples with water content from 0.000 m^3^/m^3^ to a value that is near the field capacity measured by the Hyprop for each sample. We then placed about 7.5 ml of each sample into a 15 ml stainless steel chamber. The chamber was covered with a plastic cap for 2–3 hours to allow moisture equilibration across the whole chamber. We then inserted the sample chamber into the WP4C, removed the plastic cap, and measured the *ψ* by a dew point hygrometer. Sample mass was measured to calculate *θ* [*(M*-*M*_*d*_*)V/ρ*_*w*_, m^3^/m^3^]. Based on a suite of experiments, it is difficult to prepare several samples with same *ψ*. Therefore, we used the WP4C data to estimate one *θ*_*pwp*_ (without error bars) for our biochar-sand mixtures and sand.

With Hyprop measurements we can determine *θ*_*fc*_ and WP4C measurements we can determine *θ*_*pwp*_, which then allows us to calculate plant available water (*θ*_*paw*_ = *θ*_*fc*_.- *θ*_*pwp*_).

### Bulk density and total porosity

We calculated the dry bulk density (*ρ*_*b =*_ *M*_*d*_/*V*) of each sample (sand or biochar-sand mixture) (Table 3) using measured, dry sample mass (*M*_*d*_) and total sample volume (*V* = 2.5 x 10^−4^ m^3^), which is the volume of the stainless-steel cylinder for the Hyprop.

We also calculated total porosity (*ϕ*_*T*_, volume fraction of intrapores plus interpores) (Table 3) of the biochar-sand mixtures using Eq 1.

$$\varphi_{T}=\frac{V-M_{s}/\rho_{ss}-M_{b}/\rho_{sb}}{V}$$ (1)

where *M*_*s*_ (kg) is mass of sand, *M*_*b*_ (kg) is mass of biochar, *ρ*_*ss*_ (kg/m^3^) is skeletal density of sand, and *ρ*_*sb*_ (kg/m^3^) is skeletal density of biochar (Table 2).

### Bimodal van Genuchten model

Soil water potential is composed of pressure potential, gravitational potential, osmotic potential, and perhaps by other potential terms [11], although these first three terms are understood to control most systems. Pressure potential (mainly capillary pressure) is a function of soil pore size distribution. Gravitational potential depends on the elevation reference. Osmotic potential is a function of solute concentration. Therefore, in a soil with an absence of solutes, soil water potential is controlled by soil pore size distribution.

To properly describe the water retention characteristics of our biochar-sand mixtures with interpores and intrapores, we used a bimodal van Genuchten model (VGbi) [25] to fit the measured water retention curve for each treatment.

$$\theta =\theta_{r}+(\theta_{s}-\theta_{r})\overset{k}{\underset{i=1}{\sum }}w_{i}{\left[\frac{1}{1+{\left(\alpha_{i}\left|\psi \right|\right)}^{n_{i}}}\right]}^{1-1/n_{i}}$$ (2)

In Eq 2, *θ*_*r*_ is the residual water (equal to 0 in our experiments) and *θ*_*s*_ is the saturated water content (Table 3), *k* is the number of “pore systems” (i.e. interpores and interpores) that form the total pore size distribution (total water retention curve). In our mixtures with two types of pores (interpores and intrapores), we assume that *k* is equal to 2, and *w*_*i*_ is the weighting factors of each sub soil water retention curve for each pore system where 0 < *w*_*i*_ < 1 and Ʃ*w*_*i*_ = 1. Where *α*_*i*_ (>0) is the inverse of the air entry pressure and *n*_*i*_ (>1) is a measure of the pore-size distribution the for each sub soil water retention curve, respectively. With these constraints, we simplify Eq 2 into Eq 3.

$$\theta =\theta_{s}\overset{2}{\underset{i=1}{\sum }}w_{i}{\left[\frac{1}{1+{\left(\alpha_{i}\left|\psi \right|\right)}^{n_{i}}}\right]}^{1-1/n_{i}}$$ (3)

The fitted parameters and goodness of fit were determined using the MATLAB Curve Fitting toolbox (Table 4).

**Table 4:** Bimodal van Genuchten model parameters and goodness of fit include: the weighting factors of soil water retention curve for interpores (*w*_*1*_) and intrapores (*w*_*2*_), the inverse of the air entry pressure for interpores (*α*_*1*_) and intrapores (*α*_*2*_), the measure of the pore-size distribution for interpores (*n*_*1*_) and intrapores (*n*_*2*_), R-square (*R*^*2*^) and root-mean-square error (*RMSE*).

Samples	*w*_*1*_	*α*_*1*_ (kPa^-1^)	*n*_*1*_	*w*_*2*_	*α*_*2*_ (kPa^-1^)	*n*_*2*_	*R*^*2*^	*RMSE* (m^3^/m^3^)
Fine biochar + sand	0.753	0.375	1.396	0.247	0.556	6.805	0.996	0.011
Medium biochar + sand	0.890	0.479	2.901	0.110	0.002	5.433	0.999	0.007
Coarse biochar + sand	0.849	0.458	1.601	0.151	0.010	7.740	1.000	0.003
Sand	0.914	0.415	1.568	0.086	0.013	5.790	0.998	0.006
Fine sand + sand	0.908	0.387	2.256	0.092	0.017	7.829	0.999	0.005
Coarse sand + sand	0.906	0.372	1.122	0.094	1.442	9.029	0.999	0.005

### Biochar and sand particle shape

To qualitatively examine size and shape of biochar and sand particles, we photographed biochar particles and sand particles under a microscope with a maximum zoom of 1:20 (Stereo Discovery.V20, Zeiss, Germany) (S2 Fig). To quantitatively characterize particle size and shape we used a Camsizer (Retsch Technology, Germany). We measured particle size distribution (S2 Fig) of fine, medium, and coarse biochars and sands and reported median diameter of particles’ shortest chord (*D*_*50*_) and aspect ratio (*A*_*R*_) which is *D*_*50*_ divided by median of the maximum distance between two parallel tangential lines of a particle projection (Table 5). We used these images and the Camsizer data to develop a conceptual model of how biochar particles break and mix with sand particles.

**Table 5:** Median diameter of particles’ shortest chord (*D*_*min50*_) and particles’ aspect ratio (*A*_*R*_ defined as *D*_*min50*_ divided by D_max50_) of biochar and sand used in this study. We made measurements through dynamic image analysis (Camsizer, Retsch Technology, Germany).

Materials	Sieved particle size (mm)	*D*_*min50*_ (mm)	*A*_*R*_
Fine biochar	<0.251	0.28 ± 0.0	0.61 ± 0.01
Medium biochar	0.25–0.853	0.54 ± 0.02	0.57 ± 0.03
Coarse biochar	0.853–2.00	1.41 ± 0.02	0.58 ± 0.0
Fine sand	<0.251	0.15 ± 0.01	0.74± 0.01
Sand	0.25–0.853	0.34 ± 0.01	0.73 ± 0.01
Coarse sand	0.853–2.00	1.17 ± 0.03	0.75 ± 0.01

### Statistical analyses

We used Levene’s test to confirm equality of variances of *θ*_*s*_, *θ*_*fc*_, *ρ*_*b*_, and *ϕ*_*T*_ between replicated measurements. We then performed statistical comparisons of *θ*_*s*_, *θ*_*fc*_, *ρ*_*b*_, and *ϕ*_*T*_ between different biochar-sand mixtures and sand by the one-way analysis of variance (ANOVA), followed by Tukey-Kramer’s post hoc test if differences were deemed significant at a *p*-value less than 0.05. We also computed Pearson correlation coefficients (*R*) to assess the relationships between *θ*_*s*_, *θ*_*fc*_, *θ*_*pwp*_, *θ*_*paw*_, and *ρ*_*b*_ as well as the relationships between *θ*_*s*_, *θ*_*fc*_, *θ*_*pwp*_, *θ*_*paw*_, and *ϕ*_*T*_.

## Results

Biochar grain size played an important role in the water retention of sand-biochar mixtures. Field capacity, permanent wilting point, and plant available water of sand-biochar mixtures all increased with biochar particle size.

**Fig 1:**
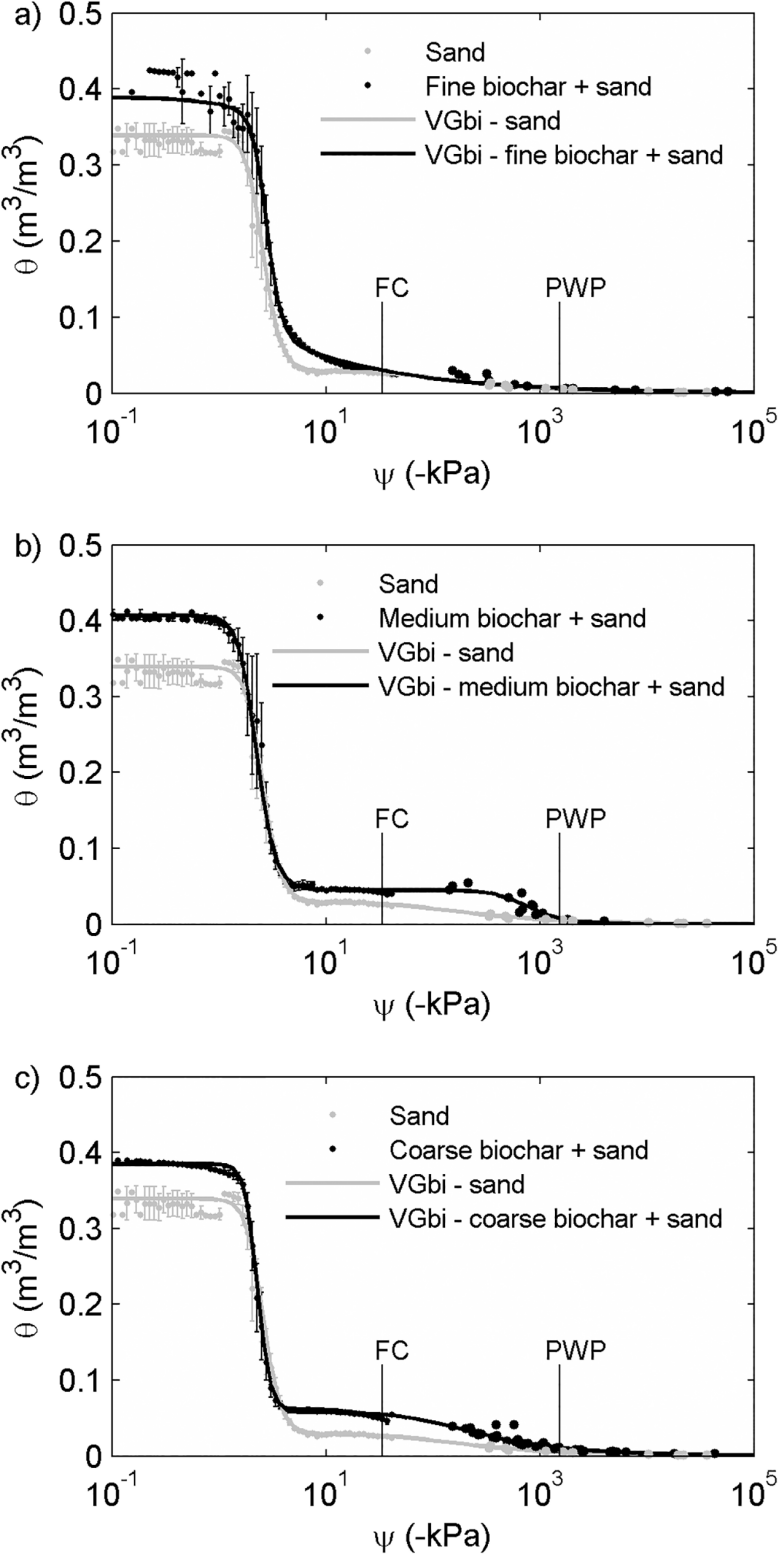
**Comparisons of water retention curves (water content, *θ*, versus soil water potential, *ψ*) between sand and sand plus 2 wt% (a) fine (b) medium and (c) coarse biochar showed that biochar addition increased water content at given soil water potential.** Data indicated with the dots were measured by the Hyprop and the WP4C and data indicated by the lines were fitted by bimodal van Genuchten model (VGbi). We report average and standard deviation of at least three measurements.

The water content at saturation (*θ*_*s*_) of fine (0.39 ± 0.03 m^3^/m^3^) and coarse (0.37 ± 0.04 m^3^/m^3^) biochar-sand mixtures were statistically the same (*p*>0.05) as that for sand (0.34 ± 0.02 m^3^/m^3^); however, the water content at saturation of the medium biochar-sand mixtures (0.41 ± 0.01 m^3^/m^3^) was 21% higher (*p*<0.01) than that for sand (Table 3). While we do not have a definitive explanation for the higher water contents at saturation in the medium biochar-sand mixture, we hypothesize that this is due to changes in packing when particles are combined of similar size, but differing aspect ratio. The differences of *θ* between biochar-sand mixtures and sand became smaller when *ψ* became lower. When *ψ* was less than -5000 kPa, water retention curves of biochar-sand mixtures and sand merged (Fig 1).

**Fig 2:**
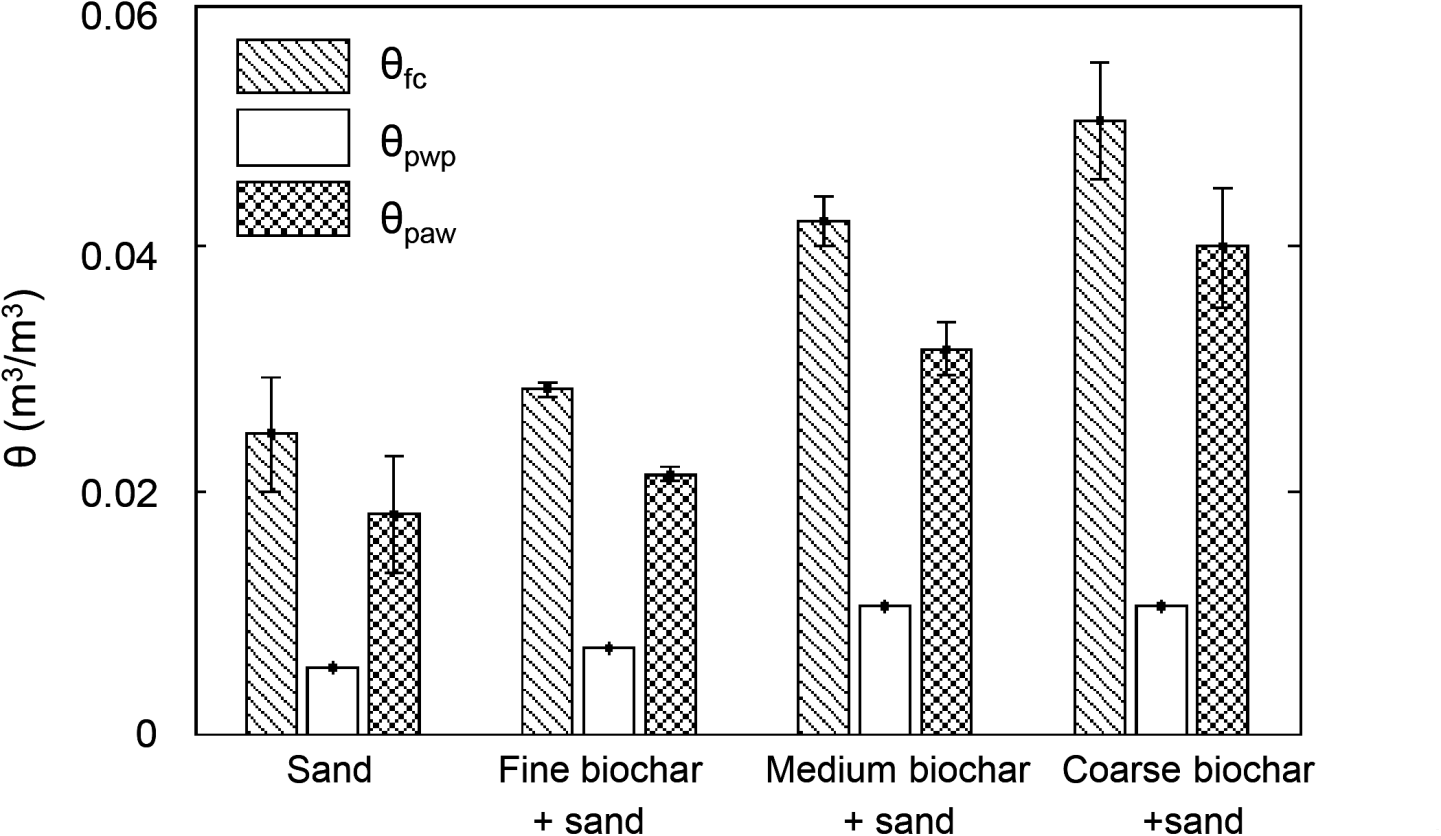
Field capacity (*θ*_*fc*_), permanent wilting point (*θ*_*pwp*_), and plant available water (*θ*_*paw*_) of sand, sand plus 2 wt% fine, medium, and coarse biochar indicated that *θ*_*fc*_, *θ*_*pwp*_, and *θ*_*paw*_ increased with biochar addition as well as biochar particle size. Values and error bars for *θ*_*fc*_ were the average and standard deviation of at least three replicates conducted for each treatment. Values for *θ*_*pwp*_ and *θ*_*paw*_ are only one replicate. Error bars of *θ*_*paw*_ are the same as error bars of *θ*_*fc*_.

Compared to the *θ*_*fc*_ of sand (0.025 ± 0.005 m^3^/m^3^), the field capacity of medium (0.042 ± 0.002 m^3^/m^3^) and coarse (0.050 ± 0.005 m^3^/m^3^) biochar-sand mixtures increased by 68% (*p*<0.01) and 100% (*p*<0.01), respectively (Fig 2). Field capacity of the fine biochar-sand mixture (0.028 ± 0.001 m^3^/m^3^) increased by 12% relative to sand but was not statistically significant (*p* = 0.25). Similarly, permanent wilting point increased from 0.005 m^3^/m^3^ for sand to 0.007 m^3^/m^3^ (40% increase), 0.010 m^3^/m^3^ (100% increase), and 0.010 m^3^/m^3^ (100% increase) for fine, medium, and coarse biochar-sand mixtures, respectively (no *p* value due to lack of replicates). These increases in *θ*_*fc*_ and *θ*_*pwp*_ resulted in increases in *θ*_*paw*_ for medium and coarse biochar-sand mixtures. Sand had *θ*_*paw*_ of 0.018 ± 0.005 m^3^/m^3^, whereas *θ*_*paw*_ was 0.021 ± 0.001 m^3^/m^3^ for fine biochar-sand mixtures (17% increase, but not different within error). Plant available water was higher for medium and coarse biochar-sand mixtures: 0.032 ± 0.002 m^3^/m^3^ (78% increase) and 0.040 ± 0.005 m^3^/m^3^ (122% increase) (Fig 2).

Compared with sand, there were no significant changes of bulk density for fine, medium and coarse biochar-sand mixtures (*p* = 0.55, 0.22, and 0.08, respectively). Total porosity increased 9.3% (*p*<0.05) for all biochar-sand mixtures (Table 3).

**Fig 3:**
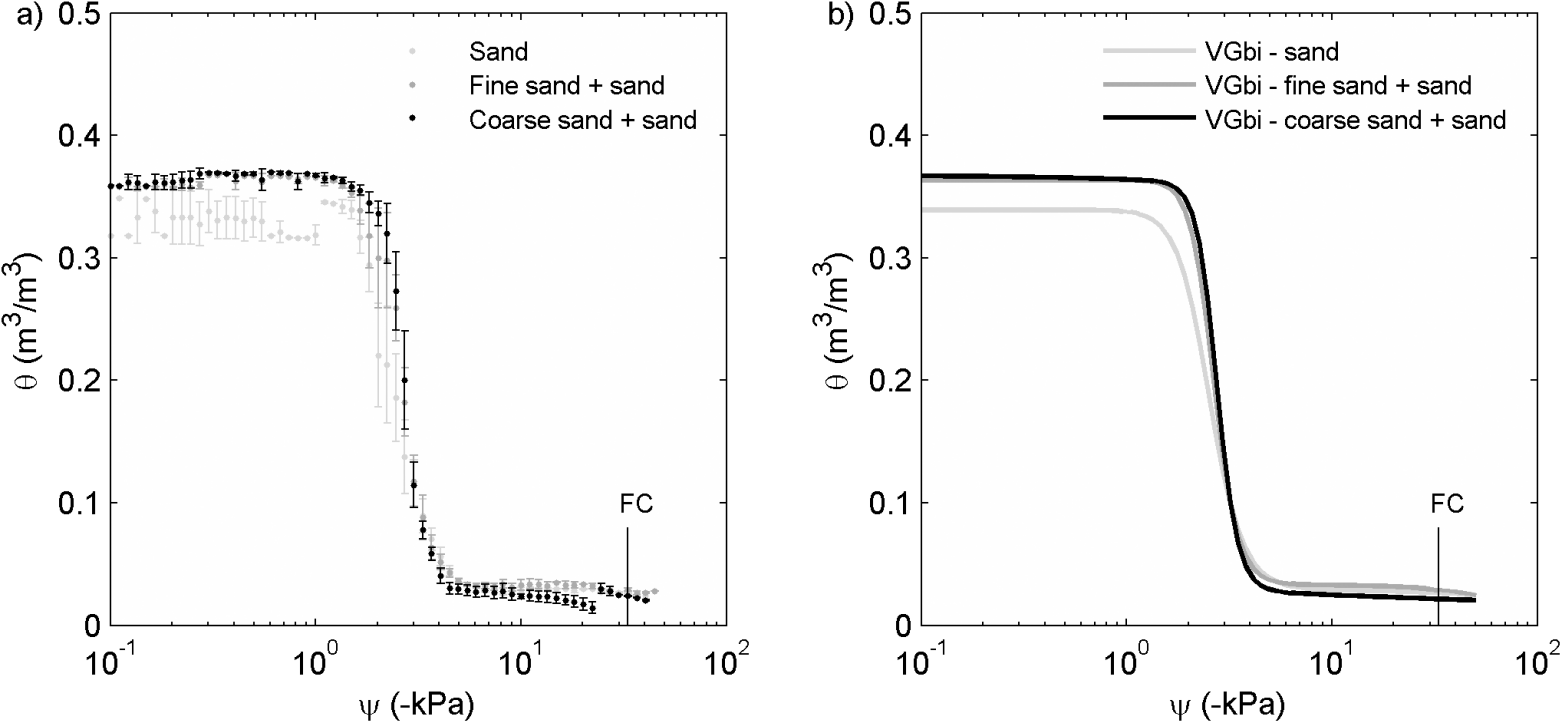
**(a). Measured water retention curves (water content, *θ*, versus soil water potential, *ψ*, measured by the Hyprop) and (b) bimodal van Genuchten model (VGbi) of data from Fig 3A.** Sand, fine sand plus sand (volume of fine sand is equal to volume of fine biochar at 2 wt% biochar rate), and coarse sand plus sand (volume of coarse sand is equal to volume of coarse biochar at 2 wt% biochar rate). These three curves overlapped with each other indicating that addition of small fraction of different sizes of sand did not cause significant change in soil water retention at such low rate.

The addition of fine sand and coarse sand increased water content at higher soil water potential values (Fig 3). This may be the result of changes in particle packing when combining sand particles of differing diameters. As water potential values dropped, the water content of sand, fine sand + sand, and coarse sand + sand mixtures became closer. At saturation the water contents for the fine sand-sand mixture (0.36 ± 0.0 m^3^/m^3^) and coarse sand-sand mixture (0.37 ± 0.01 m^3^/m^3^) were the same to that of sand alone (0.34 ± 0.02 m^3^/m^3^) within error. The water retention curves of fine sand-sand mixtures and coarse sand-sand mixtures continued to overlap with that of the sand sample for all *ψ* <-1.8kPa (Fig 3).

Compared with sand (bulk density = 1520 ± 20 kg/m^3^), the bulk density of fine sand-sand mixture (1600 ± 00 kg/m^3^) and coarse sand-sand mixture (1580 ± 30 kg/m^3^) were increased (*p*<0.05) by 5.2% and 4.0%, respectively. Correspondingly, the total porosity of the fine sand-sand mixture (0.40 ± 0.0 m^3^/m^3^) and coarse sand-sand mixture (0.43 ± 0.01 m^3^/m^3^) were 7.0% and 4.7% lower (*p*<0.05) than that of sand (0.41 ± 0.01 m^3^/m^3^).

## Discussion

Our results suggest that the pores inside biochar (intrapores) and the pores created between biochar particles and soil particles (interpores) play fundamentally different roles in soil water retention when capillarity pressure is the main component of soil water potential. Intrapores control water retention at lower soil water potential values causing an increase in field capacity, permanent wilting point, and plant available water for medium and coarse biochar-sand mixtures. However, interpores control water retention at higher soil water potential values for fine biochar-sand mixtures.

### Intrapores dominate water retention at lower soil water potential; interpores dominate water retention at higher soil water potential

To understand how pore type and size act to control soil water retention in biochar-sand mixtures, we used a simple calculation to estimate pore diameters that correlate with our observed increases in water retention. We assumed that capillary pressure (*P*_*c*_) was the major component of soil water potential.

$$\psi \approx -P_{c}=-4\gamma \cos\theta_{c}/d$$ (4)

Here *γ* is surface tension for the water-air interface at 20°C (equal to 0.072 N/m), *θ*_*c*_ (°) is contact angle between the water-air interface and biochar/sand surface and *d* (m) is the pore diameter. Contact angle describes the hydrophilicity or hydrophobicity of a solid surface [26]. The contact angle of biochar surfaces varies with pyrolysis conditions and feedstock type due to the presence of C-H functional groups on the surface of biochar particles [20]. Meanwhile, the measurement of contact angle can be affected by factors like measurement method [27, 28], liquid type [29], particle size [27], and surface morphology [30]. This leads to the complexity of measuring *θ*_*c*_ of biochar resulting in uncertainties of biochar’s *θ*_*c*_. While acknowledging these uncertainties, we assumed *θ*_*c*_ = 55° for biochar, which is the minimum reported contact angle of fresh biochar in existing studies using direct and indirect methods [7, 27–29, 31]. This assumes biochar is hydrophilic, allowing water to penetrate its intrapores. If biochar is hydrophobic (*θ* >90°), then water entry pressure is positive [32]. In this case, an applied pressure exceeding the entry pressure is needed for water to enter the biochar intrapores. Lack of this external pressure will prevent saturation of biochar intrapores. However, the hydrophobicity of biochar could be reduced by exposure to water [20], as would happen in virtually all environmental conditions, decreasing the contact angle of biochar. Therefore, we assume biochar is hydrophilic in this study, with the understanding that our results are representative of biochar that has had at least some environmental exposure.

Most biochar intrapores have diameters (*d*) <0.01 mm [33, 34]. Based on these constraints, Eq 4 provides *ψ* < -16.5 kPa when *d* is less than 0.01 mm. This suggests that the pores smaller than 0.01 mm control water retention of our biochar-sand mixtures when *ψ* is less than -16.5 kPa. Given the small size of these pores, we assume this represents intrapores (pores inside biochar particles). We did not use *θ*_*c*_ = 0° for biochar because there is unlikely that this biochar is fully hydrophillic. However, if *θ*_*c*_ = 0° for biochar, then *ψ* is equal to -28.8 kPa which is close to -16.5 kPa considering *ψ* spans several orders of magnitude.

For a mono-dispersed sand (0.251–0.853 mm), the interpore size (*d*) is larger than 0.1 mm if we assume d is 40% of particle diameter (>0.251 mm) for packed spheres [35]. We used the contact angle of a hydrophilic sand (*θ*_*c*_≈0°) because previous studies showed that biochar only causes a small degree of change in contact angle in soil [27, 28]. Based on this, we then estimated that *ψ* will be greater than -2.88 kPa when interpore size >0.1 mm. Therefore, interpores would be more likely control water retention curves at higher *ψ*.

### Biochar intrapores increase field capacity, permanent wilting point and plant available water

**Fig 4:**
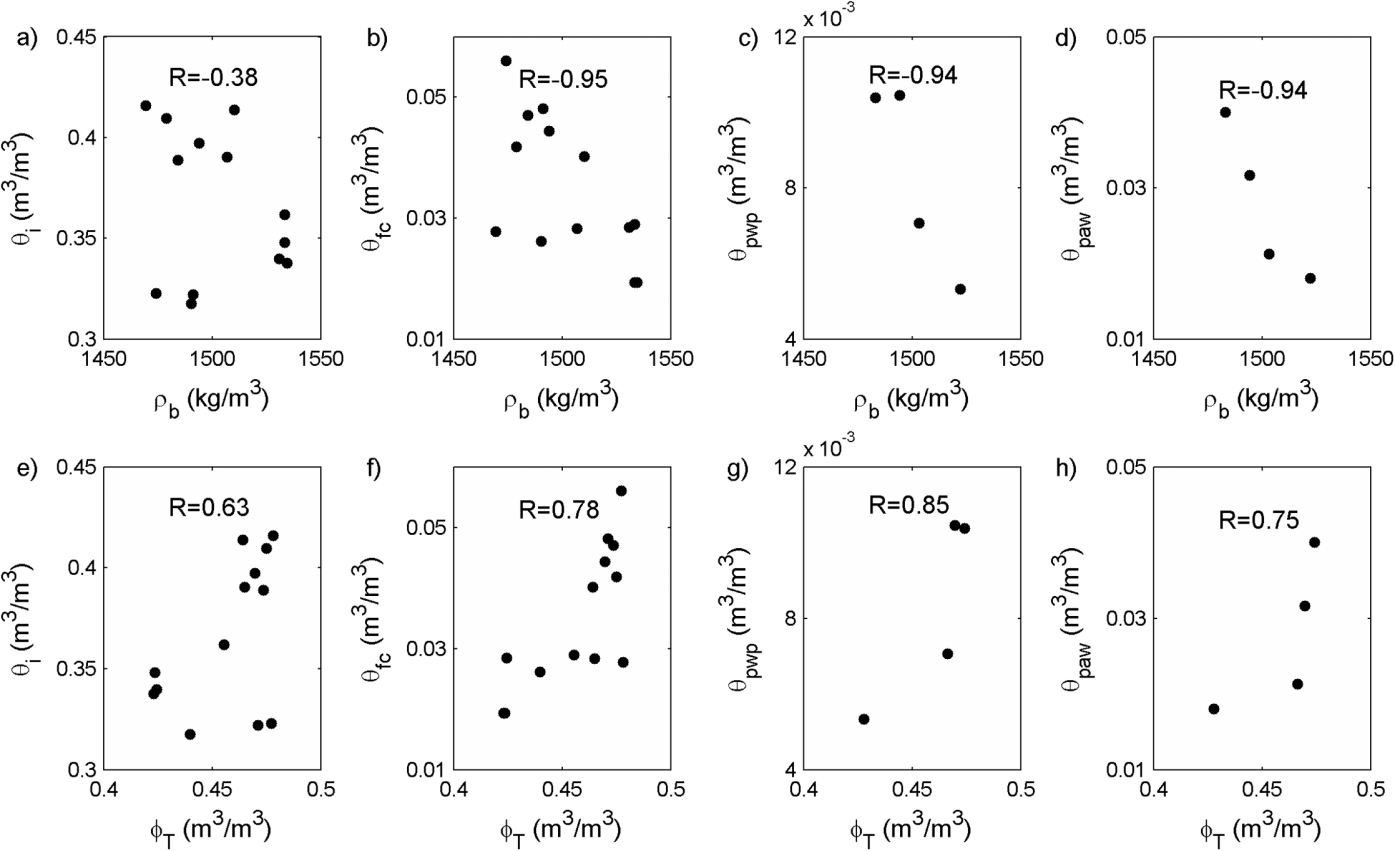
Negative Pearson correlation coefficients (*R*) between bulk density (*ρ*_*b*_) and (a) initial water content (*θ*_*i*_), (b) field capacity (*θ*_*fc*_), (c) permanent wilting point (*θ*_*pwp*_), (d) plant available water (*θ*_*paw*_) and positive *R* between total porosity (*ϕ*_*T*_) and (e) initial water content, (f) filed capacity, (g) permanent wilting point, (h) plant available water showed that soil water retention decreased with *ρ*_*b*_ increase but increased with *ϕ*_*T*_ increase.

**Fig 5:**
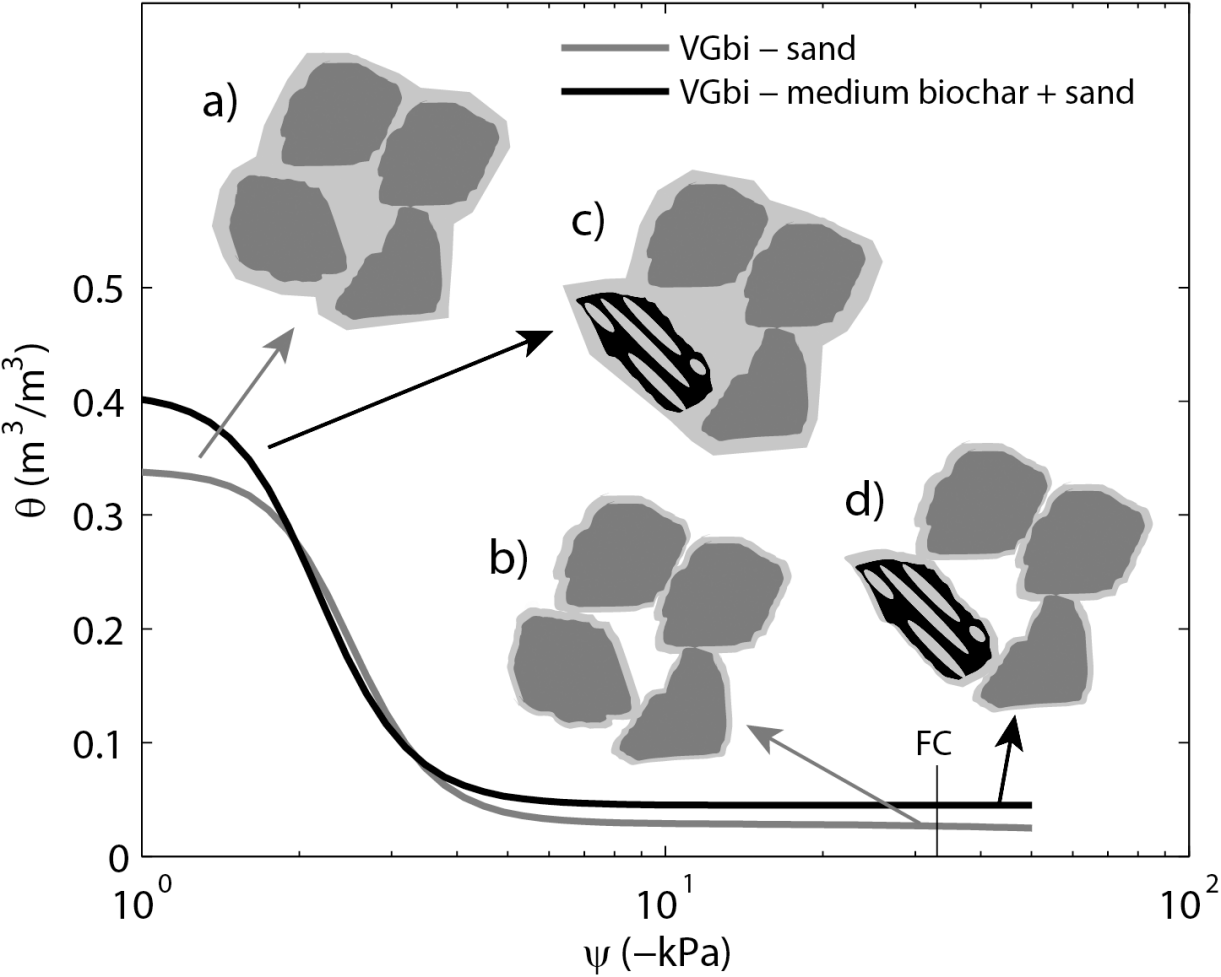
**Schematic of (a) and (b) sand (dark gray); (c) and (d) sand plus medium biochar (black) on a plot of water retention curves for these two samples.** Pores inside of biochar particles were filled with water (light gray) thus increased in water content at saturation as well as field capacity.

The high intraporosity (*ϕ*_*b*_) of the parent biochar (= 0.6 m^3^/m^3^, *ϕ*_*b*_ = 1-*ρ*_*e*_/*ρ*_*s*_) (Table 2) suggests that biochar intrapores have the capacity to increase soil water storage, and statistical analyses support this conclusion. As total porosity (*ϕ*_T_) increased, the amount of water held at a number of pressures (*θ*_*fc*_, *θ*_*pwp*_, and *θ*_*paw*_) increased for sand amended with medium and coarse biochars. We found positive relationships between *θ*_*i*_ and *ϕ*_*T*_, *θ*_*fc*_ and *ϕ*_*T*_, *θ*_*pwp*_ and *ϕ*_*T*_, *θ*_*paw*_ and *ϕ*_*T*_ (*R* = 0.63, 0.78, 0.85 and 0.75) (Fig 4). Based on these observations and our calculation that intrapores control water retention at *ψ* less than -16.5 kPa, we conclude that the increase of *θ*_*fc*_ (at *ψ* = -33kPa), *θ*_*pwp*_ (at *ψ* = -1500kPa), and *θ*_*paw*_ (= *θ*_*fc*_- *θ*_*pwp*_) (Fig 3) caused by coarser biochar addition is controlled by biochar intrapores (Fig 5).

We also found that *θ*_*fc*_, *θ*_*pwp*_, and *θ*_*paw*_ decreased as biochar particle size decreased (Fig 2). We interpret this as the result of destruction of intrapores caused by grinding biochar into smaller particles. This would decrease biochar’s internal porosity, and should be associated with an increase biochar’s skeletal density which was indeed observed (Table 2). As a result, finer biochar-sand mixtures have lower water content at a given soil water potential (Figs 1 and 2).

### How much water can biochar intrapores hold? Simple calculations to understand biochar internal water-holding capacity

We used the intraporosity of our parent biochar to calculate a realistic upper estimate of how much water biochar intrapores can hold. We then calculated the increase of water content by biochar intrapores in our experiments. By comparing these two calculations, we can better understand biochar intrapores’ actual role in water retention of soil.

The intraporosity of our parent biochar is 0.6 m^3^/m^3^, which means that the parent biochar intrapores can store up to 0.6 m^3^ water/m^3^ biochar.

The water content of biochars determined from water retention curves were lower than the water content that parent biochar can store. In section 4.1 we documented that intrapores control water retention of our biochar-sand mixtures when soil water potential (*ψ*) is less than -16.5 kPa. The water content held by medium and coarse biochars (m^3^ water/m^3^ biochar) at -16.5 kPa from measured water retention curves (*θ*_*b*_) is showed in Eq 5.

$$\theta_{b}=\frac{\theta_{diff}}{f_{vb}}$$ (5)

Where *θ*_*diff*_ (m^3^ water/m^3^ total mixture) is the difference in the water content at *ψ* = -16.5 kPa between the biochar-sand mixture and the sand, or water content increased by biochar at *ψ* = -16.5 kPa; and *f*_*vb*_ (m^3^ biochar/ m^3^ total mixture) is volume fraction of biochar which can calculated from Eq 6.

$$f_{vb}=\frac{M_{b}}{\rho_{eb}V}$$ (6)

where *M*_*b*_ (kg) is the biochar mass; *ρ*_*eb*_ (assumed to be 570 kg/m^3^ for all biochars, Table 2) is the envelope density of biochar and *V* (= 2.5 x 10^−4^ m^3^) is the sample volume of the mixture. Therefore, by combing Eqs 5 and 6, we obtained Eq 7.

$$\theta_{b}=\frac{\theta_{diff}\rho_{eb}V}{M_{b}}$$ (7)

For medium biochar, *θ*_*diff*_ = 0.016 m^3^/m^3^ and *M*_*b*_ = 7.5 x 10^−3^ kg result in a *θ*_*b*_ of 0.30 m^3^ water/m^3^ biochar. Similarly, for coarse biochar, *θ*_*diff*_ = 0.027 m^3^/m^3^ and *M*_*b*_ = 7.5 x 10^−3^ kg result in a *θ*_*b*_ of 0.52 m^3^ water/m^3^ biochar. Therefore, the amount of water (0.52 m^3^ water/m^3^ biochar) held by coarse biochar intrapores at *ψ* = -16.5 kPa is slightly less than the maximum water (0.6 m^3^ water/m^3^ biochar) that can be stored by parent biochar intrapores. However, water held by medium biochar intrapore at *ψ* = -16.5 kPa is half of the water content that can be stored by parent biochar intrapores. We interpret this to mean that the decrease in water stored in medium biochar is due to destruction of intrapores caused by grinding biochar into smaller particles.

### Biochar particle shape affects interpore volume driving changes in soil water properties

We observed that fine biochar addition to sand increased *ϕ*_*T*_ (Table 3) as well as water content for *ψ* greater than -33 kPa (Fig 1). However, there was no significant change of water content when *ψ* is less than -33 kPa. Based on our interpretation that interpores control water content when *ψ* is greater than -16.5 kPa when capillarity pressure is the main component of soil water potential, we interpret these results to mean that adding fine biochar into sand increased interpore volume.

**Fig 6:**
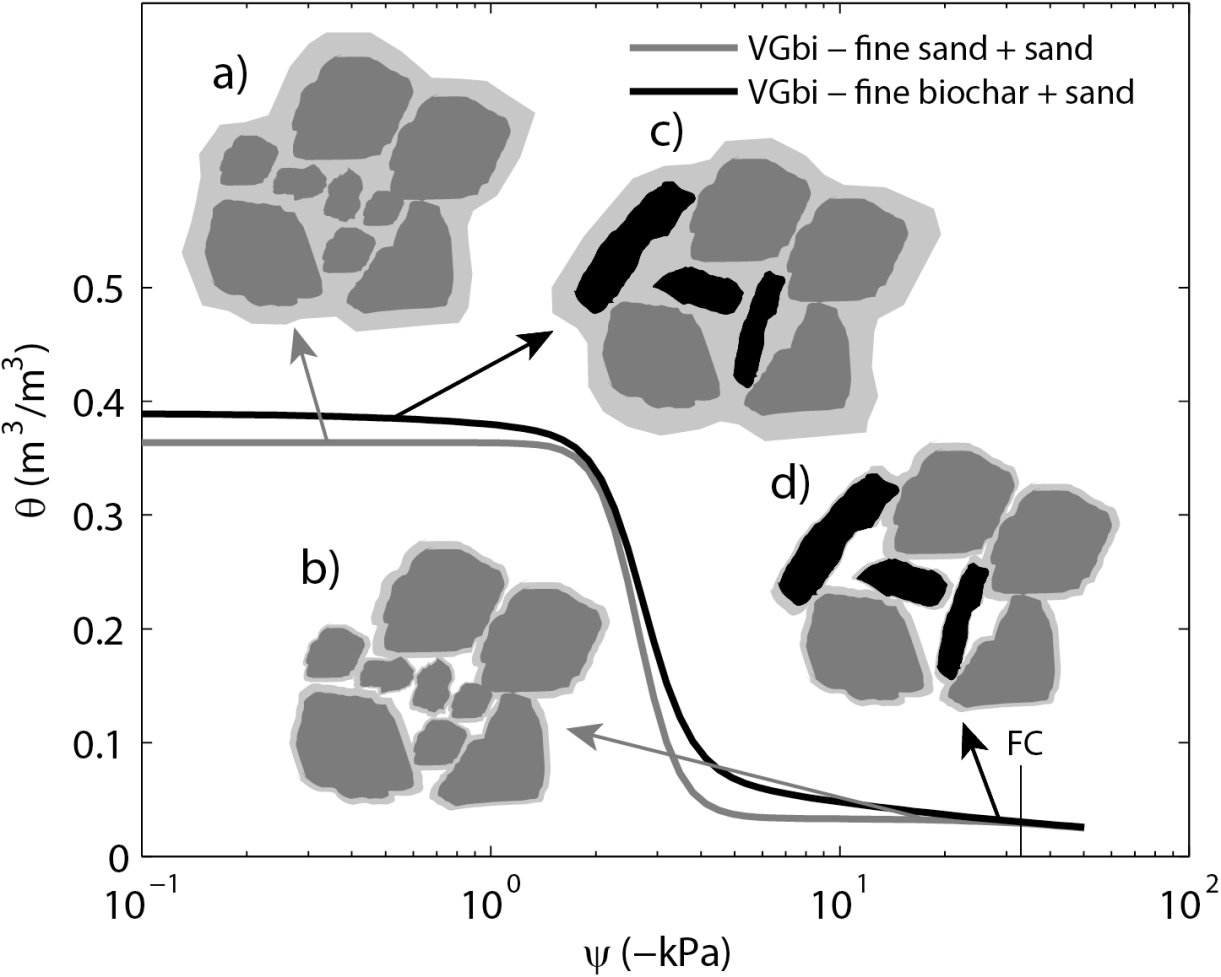
**Schematic of (a) and (b) sand (dark gray) plus fine sand and (c) and (d) sand plus fine biochar (black) on a plot of water retention curves for these two samples.** Biochar particles are more elongated which creates more pore space when packing. This may increase the distance between particles resulting in increased of interporosity. Sand plus fine biochar had a higher water (light gray shade) content than that of sand plus fine sand at higher soil water potential, probably due to its higher interporosity. However, the two water retention curves merged at lower soil water potential values (less than -33kPa) indicating that the intrapores of the fine biochar does not contribute to soil water retention as discussed in section 4.4.

Both the size and the shape of biochar particles can impact interpore volume in biochar-sand mixtures. The water retention curves of fine sand-sand mixtures and coarse sand-sand mixtures overlapped with that of sand sample for *ψ*<-2.6kPa (Fig 3). This indicated that grain size did not play an important role in this soil water potential range. However, the water content of the fine biochar-sand mixture was higher than that of the fine sand-sand mixture (volume of fine sand is equal to volume of fine biochar at 2 wt% biochar rate). Fine biochar particles were more elongated than sand particles as documented microscopic images (S1 Fig) and as quantified by lower *A*_*R*_ measured by the Camsizer (Table 5). Through numerical simulation, Deng and Davé [36] found that when elongated particles contacted each other perpendicularly or with angles other than aligned parallel, porosity increased in comparison to when all particles are spheres. Therefore, it is possible that perpendicular contacts between elongated biochar particles and sand particles created more space between particles, causing increased interporosity (Fig 6). As a result, the fine biochar-sand mixture had higher water content than the sand sample at *ψ* greater than -33 kPa. Because this soil water potential range is above field capacity, the increase of water retention is not likely to increase plant-available water under dry conditions. Instead, it would provide more storage of water on the landscape under wet conditions. For instance, the increase of *θ*_*i*_ by fine and medium biochar shows that biochar intrapores are likely to hold more water near the surface during a rain event, which may help reduce runoff. Doan et al. [37] reported that presence of biochar significantly reduced water runoff for three years of application in mesocosms. Depending on the scale of application, reduction of runoff could change local rivers’ hydrographs.

### Biochar production can be optimized to produce favorable soil water effects

Our results showed that biochar intrapores played an important role in increasing the water retention of sand-biochar mixtures water potentials less than -16.5 kPa. Water retention improvements are most useful when they impact the plant available water. We show here that intraporosity increases plant available water, suggesting that biochar with high intraporosity will be most useful. Feedstock type, pyrolysis temperature, and charring residence time influence biochar’s internal porosity [22]. For instance, biochars with low intraporosity such as wastewater sludge biochar and poultry litter biochar are less favorable for soil water storage at low water potentials (<-16.5 kPa) because their internal porosity is very low [38, 39]. Grass biochar should be better for water storage than wastewater sludge biochar because grass biochar has higher intraporosity than that of wastewater sludge biochar [22]. These interpretations are supported by existing studies. For example, Sun and Lu [40] observed an increase of plant available water by straw biochar and no effect on plant available water by wastewater sludge biochar. In their study, straw biochar increased soil pore volume of pores <10 μm but wastewater sludge biochar did not cause a significant change in soil pore volume in this size range. Higher pyrolysis temperatures produce more porous biochar [22]. Depending on feedstock type, characteristics of biochar intrapores also vary with charring residence time [41]. Therefore, an optimal charring temperature and residence time should be selected to produce biochar with high intraporosity.

The efficiency of biochar for improving soil water retention will be reduced if biochars are hydrophobic, but hydrophobicity can likely be managed by pretreatment. Hydrophobic biochar has positive water entry pressure [32], meaning that an applied force is required for water to enter intrapores. Lack of this external force would prevent water from entering intrapores thus preventing saturation of biochar intrapores and limiting water retention benefits of biochar. Jeffery et al. [31] reported that grass species biochar did not improve soil water retention; this is probably due to its high hydrophobicity (average contact angle of 118°), although it is notable that grass biochar has lower hydrophobicity compared to leaf or wood biochars [20]Biochar’s hydrophobicity varies with production temperature and feedstock [20, 29], but is usually eliminated by brief environmental exposure. Pretreating biochar either by initially wetting it, or by composting, is likely to significantly reduce problems associated with hydrophobicity [20].

### Effect of biochar on soil water retention may change over time

Our experiments documented significant increases (up to 127%) in plant-available water after mixing coarser biochar with sand at a laboratory timescale. Over the timescale of field application, biochar particle size, intraporosity, and hydrophobicity might change, likely altering soil water retention. For instance, biochar particle size can be reduced by natural forces such as freeze and thaw cycles [42], plant root penetration [43], and bioturbation [44]. Biochar’s intraporosity can also be reduced by sorption of minerals [45, 46], adsorption of organic matter [47], and microorganism growth [48]. Using x-ray photoelectron spectroscopy, LeCroy et al. [49] found evidence of increased surface oxidation on biochar particles suggesting that the first stage of biochar patina development involves sorption of dissolved organic compounds in soil. In addition, microscopic and pycnometric data from recent field trials point to blockage of biochar intrapores by either organics, minerals, or a combination [46]. Biochar hydrophobicity can prevent water from penetrating into biochar intrapores, prohibiting an improvement of soil water retention [31]. However, Ojeda et al. [27] observed a 69.5% decrease of contact angle of biochar after one year of its addition to soil suggesting that initial biochar hydrophobicity disappeared within one year. This decrease in hydrophobicity will improve soil water retention.

## Conclusion

In this study, we used a simple sand-biochar system to develop a mechanistic understanding of how biochar’s internal pores (intrapores) and the pores between biochar and sand particles (interpores) affect soil water retention. In our experiments the addition of biochar to sand increased initial water content and field capacity. Our controlled particle size and porosity conditions allowed the development of conceptual models that connect biochar properties to soil water benefits. We propose that the increase of water retention of sandy soils by biochar addition is caused by biochar intraporosity at lower *ψ* and by increasing interporosity due to elongated biochar particle shape increasing interpores space at higher *ψ* when capillarity pressure is the main component of soil water potential. This suggests that to increase plant-available water (*θ*_*paw*_) in sandy soils, biochar with high intraporosity and an irregular shape will be most effective. Various production factors (i.e. feedstock, pyrolysis temperature and charring residence time) may be useful to produce biochar with varying porosity. Further studies are needed to address how long biochar intraporosity, particle size, and particle shape will last after field application.

## Supporting information

S1 Fig Photomicrograph of (a) fine biochar (<0.251 mm), (b) fine sand (<0.251 mm), (c) medium biochar (0.251–0.853 mm), (d) sand (0.251–0.853 mm), (e) coarse biochar (0.853–2.00 mm), (f) coarse sand (0.853–2.00 mm) and (g) parent biochar (2.00–2.30 mm).
(TIF)

S2 Fig Particle diameter (D) distribution (% by volume) of materials used in this study, as measured by a Retsch Camsizer. Particle diameter (*D*) is the shortest chord of a particle projection (results close to screening/sieving).
(TIF)

S1 Table Nomenclature describes all symbols used in this study. (XLS)
