# Supplementary figures and images for: Biochar particle size, shape, and porosity act together to influence soil water properties
Source: PLoS One. 2017 Jun 9;12(6):e0179079. doi: 10.1371/journal.pone.0179079 (PMC5466324; doi:10.1371/journal.pone.0179079)

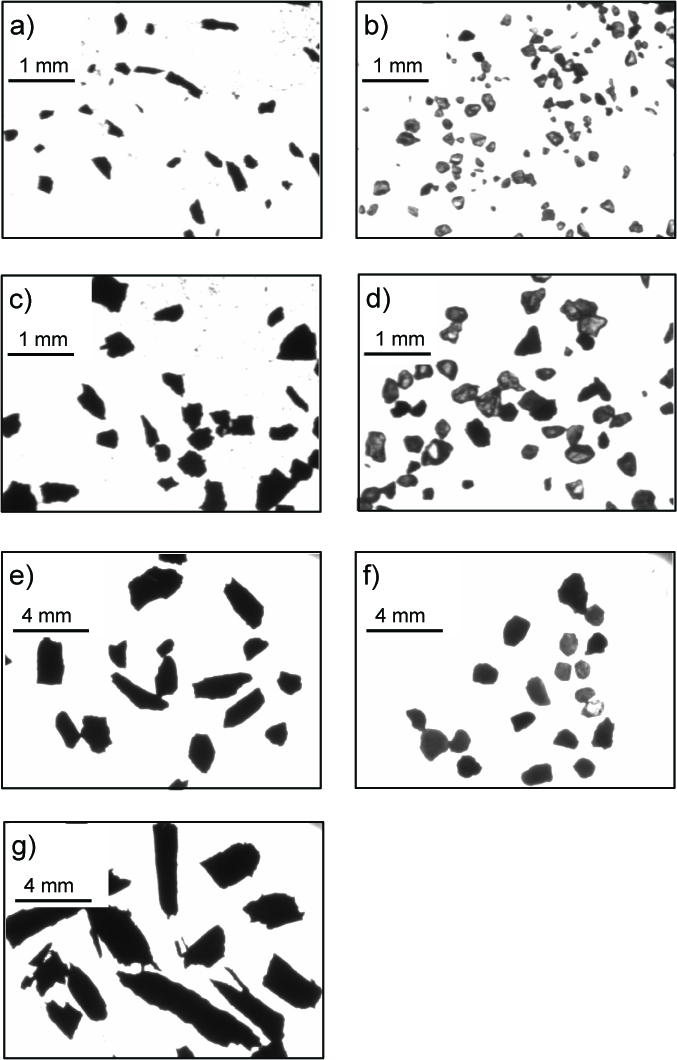

Supplement: S1 Fig — Photomicrograph of (a) fine biochar (<0.251 mm), (b) fine sand (<0.251 mm), (c) medium biochar (0.251–0.853 mm), (d) sand (0.251–0.853 mm), (e) coarse biochar (0.853–2.00 mm), (f) coarse sand (0.853–2.00 mm) and (g) parent biochar (2.00–2.30 mm). (TIF) [file pone.0179079.s001.tif]

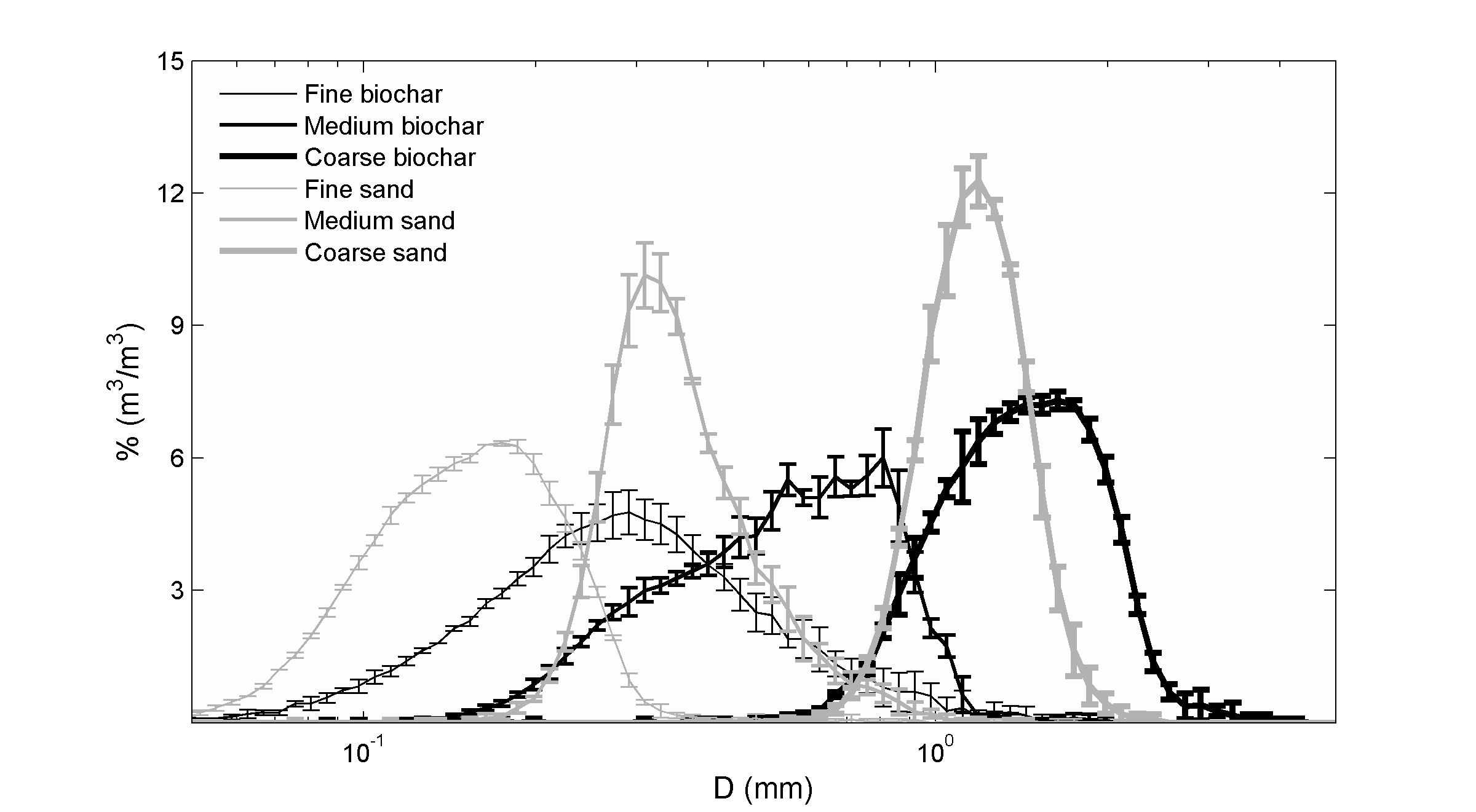

Supplement: S2 Fig — Particle diameter (D) is the shortest chord of a particle projection (results close to screening/sieving). (TIF) [file pone.0179079.s002.tif]
